# Pulmonary exacerbations in early cystic fibrosis lung disease are marked by strong modulation of CD3 and PD-1 on luminal T cells

**DOI:** 10.3389/fimmu.2023.1194253

**Published:** 2023-09-21

**Authors:** Vincent D. Giacalone, Diego Moncada Giraldo, George L. Silva, Justin Hosten, Limin Peng, Lokesh Guglani, Rabindra Tirouvanziam

**Affiliations:** ^1^ Department of Pediatrics, Emory University, Atlanta, GA, United States; ^2^ Center for CF and Airways Disease Research, Children’s Healthcare of Atlanta, Atlanta, GA, United States; ^3^ Wallace H. Coulter Department of Biomedical Engineering, Georgia Institute of Technology and Emory University, Atlanta, GA, United States; ^4^ Department of Biostatistics and Bioinformatics, Emory University School of Public Health, Atlanta, GA, United States

**Keywords:** cystic fibrosis, cytokines, exacerbation, inflammation, neutrophils, T cells

## Abstract

**Background:**

In chronic cystic fibrosis (CF) lung disease, neutrophilic inflammation and T-cell inhibition occur concomitantly, partly due to neutrophil-mediated release of the T-cell inhibitory enzyme Arg1. However, the onset of this tonic inhibition of T cells, and the impact of pulmonary exacerbations (PEs) on this process, remain unknown.

**Methods:**

Children with CF aged 0-5 years were enrolled in a longitudinal, single-center cohort study. Blood (n = 35) and bronchoalveolar lavage (BAL) fluid (n = 18) were collected at stable outpatient clinic visits or inpatient PE hospitalizations and analyzed by flow cytometry (for immune cell presence and phenotype) and 20-plex chemiluminescence assay (for immune mediators). Patients were categorized by PE history into (i) no prior PE, (ii) past history of PE prior to stable visit, or (iii) current PE.

**Results:**

PEs were associated with increased concentration of both pro- and anti-inflammatory mediators in BAL, and increased neutrophil frequency and G-CSF in circulation. PE BAL samples showed a trend toward an increased frequency of hyperexocytic “GRIM” neutrophils, which we previously identified in chronic CF. Interestingly, expression levels of the T-cell receptor associated molecule CD3 and of the inhibitory programmed death-1 (PD-1) receptor were respectively decreased and increased on T cells from BAL compared to blood in all patients. When categorized by PE status, CD3 and PD-1 expression on blood T cells did not differ among patients, while CD3 expression was decreased, and PD-1 expression was increased on BAL T cells from patients with current PE.

**Conclusions:**

Our findings suggest that airway T cells are engaged during early-life PEs, prior to the onset of chronic neutrophilic inflammation in CF. In addition, increased blood neutrophil frequency and a trend toward increased BAL frequency of hyperexocytic neutrophils suggest that childhood PEs may progressively shift the balance of CF airway immunity towards neutrophil dominance.

## Introduction

Pulmonary exacerbations (PEs) are a frequent occurrence among people with CF, with up to 20,000 episodes recorded in the United States each year in a population estimated at 40,000 (1). PEs are characterized by increased cough, mucus production and reduced lung function and are often promoted by pulmonary infections; with severe PEs requiring treatment with intravenous antibiotics (2). PEs can be detrimental even for young children with CF who do not yet exhibit overt structural lung damage, as repeated PEs have been shown to accelerate lung function decline (3). Pro-inflammatory cytokine secretion has been reported in numerous studies as a consequence of PEs (4–6), particularly the potent neutrophil chemoattractant CXCL8 (also known as interleukin-8) (7, 8). Total circulating leukocyte counts are elevated during PEs and have been shown to decrease following antibiotic treatment (7) but little is known about phenotypic or functional changes that immune cells undergo in the airway during PEs. Also, there are key limitations to prior studies of inflammatory markers associated with PEs, including discrepancies in criteria used to define PEs in different centers, differences in sample collection and processing procedures, and the time at which samples are collected between the advent of PEs and the inception of high-impact treatment (9).

As CF lung disease progresses during childhood, infiltrating neutrophils become the dominant cell type in the airway lumen where they undergo functional reprogramming including increased degranulation, and nutrient transporter expression, but decreased expression of phagocytic receptors (10). We termed these highly activated airway-adapted neutrophils “GRIM” (for granule releasing, immunomodulatory, and metabolically active) (11). Previously, we demonstrated that release of neutrophil elastase (NE) by GRIM neutrophils in CF lungs correlates with measures of lung disease (structural damage by chest computed tomography and lung function by spirometry). We also showed that GRIM neutrophils suppress other immune cell populations in CF airways, including macrophages via activation of the inhibitor PD-1 pathway (12) and T cells via secretion of arginase 1 (Arg1) from the primary granules, which cleaves the essential amino acid arginine required by T cells for T-cell receptor signaling (13). Other granule enzymes such as NE and matrix metalloproteinases are major contributors to development of bronchiectasis (14). Despite the highly inflammatory state of the airways, people with CF are unable to clear routine infections (15), due in large part to reduced bacteria killing capability of these GRIM neutrophils (16). Despite an increased understanding of the pathological adaptations exhibited by neutrophils in the CF airways and the mechanisms through which they modulate the function of other leukocyte populations, we do not fully understand the sequence of how these events occur or how they may be impacted by childhood events.

Early life PEs may offer the opportunity to study non-neutrophil populations in the airway, before neutrophils dominate this microenvironment and alter their activity. Furthermore, these events may prefigure impending neutrophilic inflammation, since recent PE occurrence is the most important risk factor for occurrence of future PEs (17). To gain further understanding of both soluble and cellular markers of inflammation related to PEs, we obtained blood and bronchoalveolar lavage (BAL) samples from young children with CF at stable clinic visits and hospitalizations for treatment of a PE. Some of these subjects enrolled for collection of samples at stable clinic visits had prior hospitalizations for a PE, but all subjects in the study were in the early stages of CF lung disease. Through measurement of immune mediators, NE, and leukocyte phenotypes in blood and BAL, we demonstrate that early life PEs are not characterized by elevated biomarkers of neutrophilic inflammation in the airway. Rather, early life PEs associate with changes in neutrophil poise in the circulation, which may foreshadow future neutrophilic inflammation in the airways. Furthermore, we show evidence for T-cell activation during early-life PEs, which differs from later stages of CF lung disease when T cells in the airways are strongly inhibited by neutrophils (13).

## Materials and methods

### Human subjects and samples

Twenty-six patients (median age at enrollment = 35 months, age range 23 months - 8.5 years, male:female ratio = 2.3) with CF were enrolled over a period of 3 years as part of the IMPEDE-CF study at Emory University and Children’s Healthcare of Atlanta. All aspects of subject enrollment and sample collection were approved by the Emory University Institutional Review Board (IRB00097352). A total of 39 study visits were conducted on these subjects with collection of blood and/or BAL samples at the clinic during a scheduled stable visit or in the hospital upon admission for a PE.

Visits were classified into three groups based on their history of PE as follows: (i) no PE: samples collected at a stable clinic visit, from a subject with no history of hospitalization for treatment of PE; (ii) prior PE: samples collected at a stable clinic visit from a subject who had previous history of having been hospitalized for treatment of a PE; (iii) current PE: samples collected during current hospital admission for a PE.

A summary of demographics for the 39 study visits grouped by PE status is presented in **Table 1**. Detailed subject demographics (including but not limited to race/ethnicity, mutation types, BAL microbiology) are provided in **Table S4**. As is common for pediatric studies, not all samples/outcomes could be obtained for all visits, due to prioritization of samples for clinical testing and limitations in material available for research. Sample collection totals are recorded in **Table S1**, with a summary of assays performed provided in **Table S2**.

**Table 1:** Demographics of enrolled subjects.

	Sample collection				Sex		CFTR mutation			Modulators	
PE status	Clinic visits	Median age (yrs)	Blood	BAL	M	F	HO	HZ	OT	IVA	LUM+ IVA
No PE	18	3.5	17	7	10	8	8	9	1	0	1
Prior PE	13	2.92	11	6	11	2	4	5	4	1	1
Current PE	8	3.25	6	5	7	1	5	3	0	0	0

A total of 34 blood samples and 18 BAL samples were collected at 39 clinic visits. Subjects were divided by PE history, including stable clinic visit with no history of exacerbation (no PE), stable clinic visit but previously hospitalized for PE (prior PE), and currently hospitalized for treatment of PE (current PE). F, female; HO, homozygous for the F508del mutation; HZ, heterozygous for the F508del mutation; IVA, ivacaftor; LUM, lumacaftor; M, male; OT, carrying two mutations other than F508del.

### Blood and BAL collection and processing

Blood was collected by venipuncture using K2 EDTA tubes, and were processed within 2 hours of collection by centrifugation at 400 x g (10 minutes, 4°C) to separate plasma from blood cells. The plasma was removed and further centrifuged at 3000 x g (10 minutes, 4°C) to remove platelets and debris. The platelet and debris-free plasma was frozen at -80°C and banked for later quantification of immune mediators. The blood cell pellet was washed with 10 mL of sterile ice-cold phosphate-buffered saline with 2.5 mM EDTA added (PBS+EDTA). After centrifugation at 400 x g (10 minutes, 4°C) and aspiration of supernatant, the blood cell pellet was resuspended to its original volume using PBS+EDTA. This washed blood pellet was used for staining and flow cytometry analysis of blood leukocytes.

BAL was performed during flexible fiberoptic bronchoscopy procedures under general anesthesia at stable visits and hospitalizations for PEs. A 3.1 mm bronchoscope (BF-XP190, Olympus, Japan) with a 1.2 mm suction channel was used to retrieve BAL fluid. All BAL samples were collected from the right middle lobe by instillation and aspiration of sterile 0.9% saline (1 mL/kg up to a maximum volume of 20 mL per aliquot). A total of 2 or 3 aliquots were instilled depending on the yield, with the first aliquot reserved for clinical microbiology. Microbiological cultures and cytology typically require 7 mL of BAL fluid, with additional volumes transported on ice to the research laboratory for immediate processing. EDTA was added to each BAL sample for a final concentration of 2.5 mM. Blood and BAL sample yields are summarized in **Table S1**. BAL samples were processed as described previously (18). In brief, the sample was homogenized by passing through an 18-gauge needle for 12 cycles. The sample was centrifuged at 800 x g (10 minutes, 4°C) and the supernatant was removed for a further centrifugation at 3000 x g (10 minutes, 4°C) to yield debris-free supernatant. The BAL cell pellet was washed in PBS+EDTA and counted using fluorescent microscope with ethidium bromide + acridine orange staining. BAL supernatants were frozen at -80°C and banked for later quantification of immune mediators and NE, and cells were stained for analysis by flow cytometry.

### Immune mediators

A 20-plex panel of inflammatory response mediators was measured in plasma and BAL using a high-sensitivity chemiluminescence assay (U-PLEX, Meso Scale Diagnostics) according to the manufacturer’s protocol. Analytes included mediators related to neutrophil recruitment/activation (CXCL1, CXCL5, CXCL8, CXCL10, CXCL11, TNF-α), monocyte/macrophage recruitment/activation (CCL2, CCL4, IFN-γ, IL-6, VEGF-A), the IL-1 family (IL-1α, IL-1β, IL-18), the colony-stimulating factor (CSF) family (G-CSF, M-CSF, GM-CSF), and anti-inflammatory mediators (IL-1RA, IL-10, TNFSF10). We note that some mediator may be bound to factors that could impede their measurement, e.g., IL-18 binding to IL-18BP. In this particular case, proprietary data from the vendor (Meso Scale Diagnostics) confirmed that IL-18 detection is not impaired by binding to other factors such as IL-18BP. It is important to note that these are generalized groupings and individual mediators may have different functions or classifications in specific contexts. For concentrations that fell below the lower limit of detection, a value of half the lower limit was assigned. These values are represented as open symbols in related figures. Statistical comparisons were not performed between groups if more than half of the data points from one group consisted of imputed values.

### NE activity

Extracellular NE activity in BAL was measured by Förster resonance energy transfer (FRET) assay using the NEmo‐1 probe (Sirius Fine Chemicals SiChem GmbH), as previously described (19–21).

### Flow cytometry

Analysis of leukocytes in blood and BAL fluid by flow cytometry was performed as described previously, using a gating strategy to identify neutrophils, monocytes/macrophages, and T cells (18). The gating strategy with representative subject-matched blood and BAL is shown in **Figure S2**. In brief, cells were pre-stained with Fc block to prevent non-specific binding of antibodies (Biolegend #422302) and calcein violet for viability for 10 minutes, followed by staining with antibodies for surface proteins for 20 minutes. Targets included CD3, CD16, CD36, CD41a, CD45, CD63, CD66b, CD115, CD304, epidermal growth factor receptor (EGFR), surface NE (R&D Systems), PD-1, PD-L1, and Siglec-8. All incubations were performed on ice in the dark. Cells were washed with PBS+EDTA and fixed in BD Phosflow Lyse/Fix Buffer (BD Biosciences #558049) by incubating overnight in the dark at 4°C. The next day fixed cells were washed with PBS+EDTA and stored at 4°C in the dark until acquisition. Samples were acquired in batches when possible but all were acquired within 2 weeks of staining. All samples were acquired on a BD LSRII or BD FACSymphony, which were calibrated using 6-peak Rainbow Calibration Particles (Biolegend #422901), as previously described (22), to ensure consistent fluorescence output. Compensation was computed using single-stained UltraComp eBeads (Invitrogen #01-2222-42). All compensation, gating, and calculation of median fluorescence intensity (MFI) and cell frequencies was performed using FlowJo V9.9.5 (BD). A threshold of at least 50 events after all gating steps was established for populations to be considered reliable for analysis of fluorescence parameters, as we demonstrated before in published analyses of blood, sputum and BAL (12, 18, 22, 23). Staining panels are detailed in **Table S4**.

### Statistical analysis

Data were analyzed in Prism (version 8; GraphPad Software) to conduct group comparisons, using nonparametric statistical tests (Mann-Whitney) due to the small sample size. Principal component analysis (PCA) was conducted using MATLAB.

## Results

### Participant demographics

Of the 39 study visits conducted, 28 were on male and 11 on female subjects (72% and 28%, respectively). Breakdown based on F508del mutation status was 17 for homozygotes, 17 for heterozygotes, and 4 for other mutations (44%, 44% and 12%, respectively). The median age at the time of the study visit was 35 months. On review of their medical records, 19 visits (49%) were on subjects who had no prior history of any hospitalizations for PEs, 12 (31%) on subjects who had one or more prior hospitalizations and 8 on subjects currently hospitalized for PE (49%, 31% and 20%, respectively). Only a limited number of visits were on subjects on CFTR modulator therapy (1 on ivacaftor, and 2 on lumacaftor + ivacaftor) due to age and approved therapies available at the time. Of the 8 visits on subjects with current PE, 5 had viral testing done within 48 hours of admission, with 3 of them being positive for a viral infection (parainfluenza 4, rhinovirus and influenza A virus infections). The average total length of hospital stay for subjects with current PE was 10.1 days.

### Current PEs are associated with pulmonary infections

We assigned each study visit to one of three groups based on PE history as described in Methods, including no PE, prior PE, and current PE. We then compared airway colonization by pro-inflammatory pathogens and neutrophil frequency in BAL among these groups. In this cohort, detectable pro-inflammatory pathogens included *S. aureus* and *S. marcescens* (24), listed in **Table S4**. Study visits in the no PE and prior PE groups were evenly divided between infected and non-infected (10 detected vs. 9 not detected, and 5 detected vs. 7 not detected, respectively), while the majority of visits for subjects currently experiencing a PE had detectable airway pathogens (6 detected vs. 2 not detected). We then compared airway neutrophilia in each group of subjects, using 10% as the threshold for determining elevated neutrophil frequency in BAL. Neutrophil frequencies were greater than 10% in all samples except for one BAL sample from the no PE group (**Table 2**; **Figure S1**).

**Table 2:** PEs are associated with airway infection.

	Infection status		Neutrophil frequency in BAL	
PE status	Uninfected	Infected	<10%	>10%
No PE	9	9	1	5
Prior PE	7	6	0	6
Current PE	2	6	0	4

Infections were defined as 2 out of 4 positive throat swab cultures with a pro-inflammatory pathogen. Neutrophil frequency in BAL was determined by flow cytometry. Study visits were divided by PE history, including stable clinic visit with no history of PE (No PE), stable clinic visit but previously hospitalized for PEs (Prior PE), and currently hospitalized for treatment of an PE (Current PE).

### Pro- and anti-inflammatory immune mediators are elevated in BAL and plasma during early CF PEs

**Figure 1:**
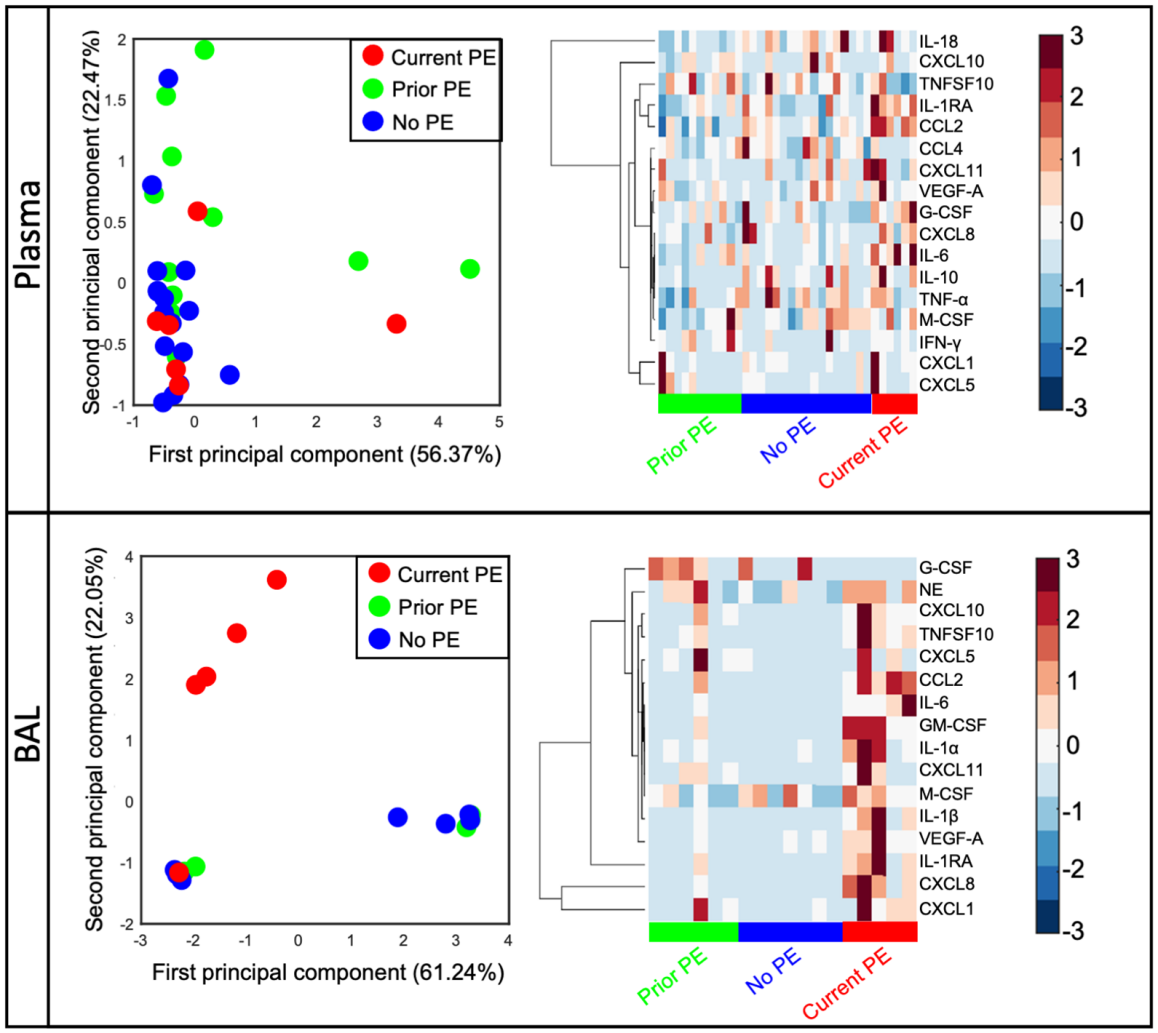
CXCL1 and CXCL8 are distinguishing signatures of early CF PEs. Principal component analysis of immune mediators in plasma and BAL from no PE (n = 17 in plasma, 7 in BAL), prior PE (n = 11 in plasma, 6 in BAL), and current PE (n = 6 in plasma, 5 in BAL) groups shows separation of the latter in BAL (but not plasma) which is driven by CXCL1 and CXCL8.

Previous studies have shown inflammatory mediators to be elevated in response to PEs and declining after treatment with antibiotics (4–8). However, much of these data are from adolescent or adult subjects with very little data from young children with early stages of CF lung disease. We quantified 20 immune mediators in BAL and plasma and compared concentrations between study visits from no PE, prior PE, and current PE categories. First, we conducted an unsupervised clustering by PCA in plasma and BAL. We excluded GM-CSF, IL-1α, and IL-1β in plasma and CCL4, IFN-γ, IL-10, IL-18, and TNF-α in BAL due to many data points below the limit of detection. In this PCA analysis, data points representing individual subjects will cluster together if they share similar cytokine profiles. In plasma, we observed no separation of visits according to no PE, prior PE, and current PE categories based on immune mediator concentrations in each group (**Figure 1**). However, in BAL, current PE visits were distinct from no PE and prior PE groups, with the neutrophil chemoattractants CXCL1 and CXCL8 driving this distinction by PCA as shown in the accompanying clustergram (**Figure 1**).

**Figure 2:**
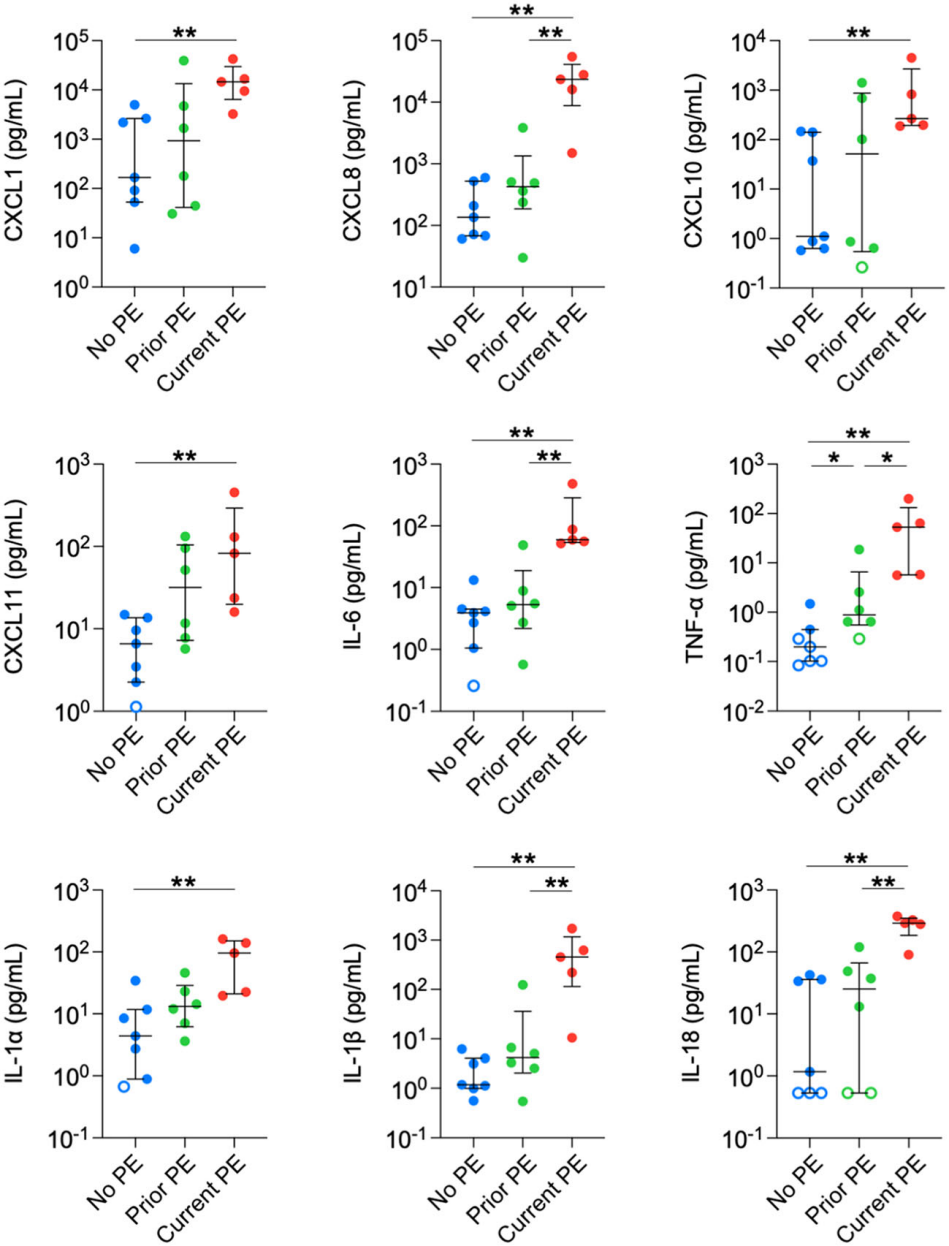
Neutrophil chemoattractants are elevated in BAL during PEs. Immune mediators in BAL were compared between no PE (n = 7), prior PE (n = 6), and current PE (n = 5) groups using the Mann-Whitney test. An imputed value of ½ the lower limit of detection (LLOD) was assigned for data points < LLOD (open symbol). Significant differences are indicated as *p ≤ 0.05, **p ≤ 0.01.

Next, we compared concentrations in BAL between no PE, prior PE, and current PE groups. Nine neutrophil-related mediators were significantly elevated in the current PE group compared to the no PE group, namely CXCL1, CXCL8, CXCL10, CXCL11, IL-6, TNF-α, IL-1 α, IL-1β, and IL-18. In addition, CXCL8, IL-6, TNF-α, IL-1β and IL-18 were significantly higher in the current PE group vs. prior PE group, while only TNF-α was greater in the prior PE group vs. the no PE group (**Figure 2**).

**Figure 3:**
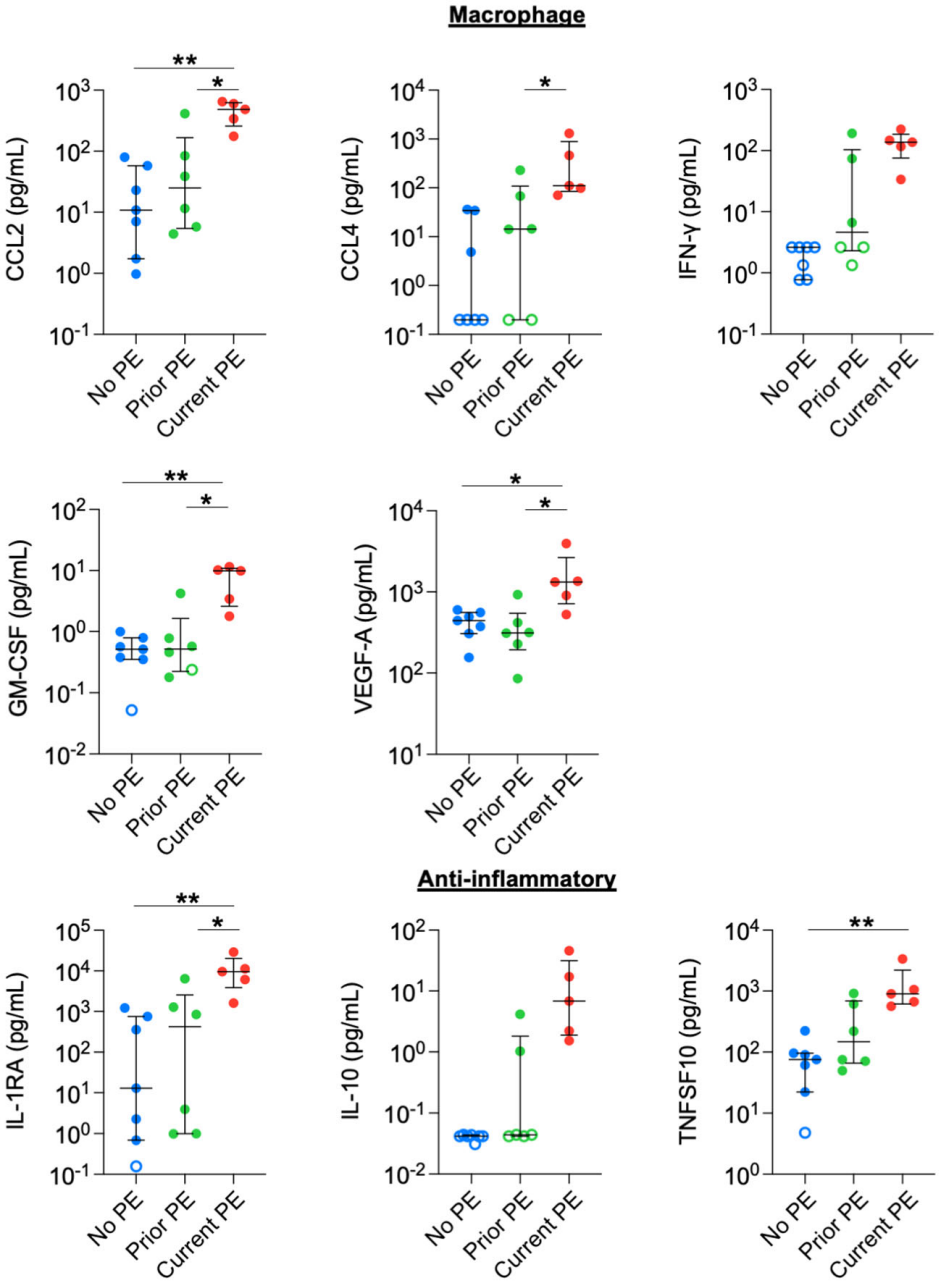
Macrophage-related and anti-inflammatory cytokines are elevated in BAL during PEs. Immune mediators in BAL were compared between no PE (n = 7), prior PE (n = 6), and current PE (n = 5) groups using the Mann-Whitney test. An imputed value of ½ the LLOD was assigned for data points < LLOD (open symbol). Statistical comparison was not performed between groups for IFN-γ or IL-10, where the majority of points were < LLOD. *p ≤ 0.05 and **p ≤ 0.01.

We also observed increased concentrations in monocyte/macrophage-related and anti-inflammatory cytokines. CCL2, GM-CSF, and VEGF-A were higher in the current PE group compared to the no PE and prior PE groups. CCL4 levels were also higher in the current PE group vs prior PE group, and also comparing the current PE group vs. no PE group, although the latter was not compared statistically due to the majority of data points for the no PE group being below the limit of detection for CCL4. Similarly, the concentration of IFN-γ was higher in the current PE group than in no PE and prior PE groups, but this was not compared statistically due to points from the latter two groups below the limit of detection for IFN-γ (**Figure 3**). Among anti-inflammatory mediators, IL-1RA was higher in the current PE group compared to no PE and prior PE groups. Same was true of IL-10, which was not compared statistically due to points below the limit of detection in no PE and prior PE groups. TNFSF10 was also higher in the current PE group vs. the no PE group (**Figure 3**).

**Figure 4:**
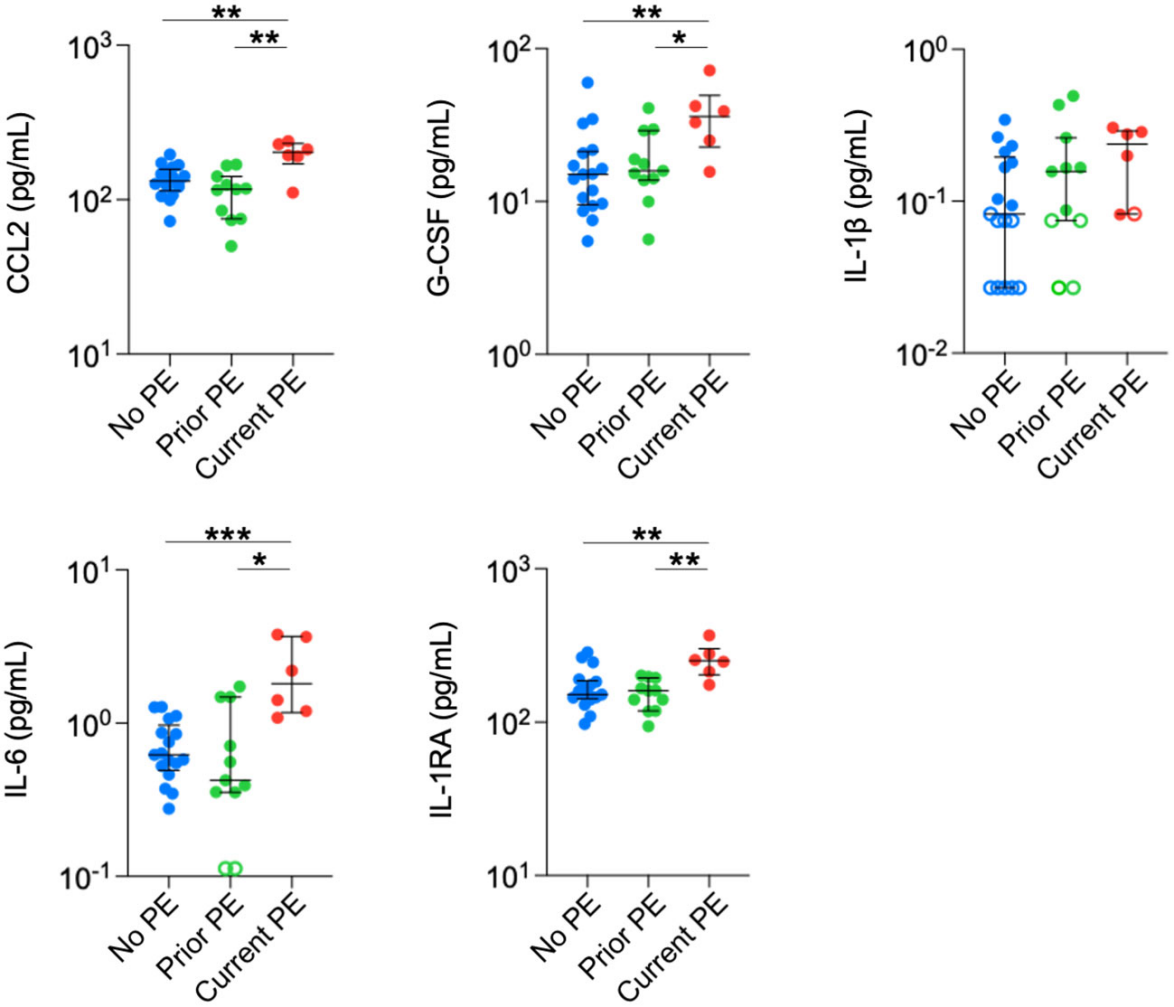
Neutrophil chemoattractants and anti-inflammatory cytokines are elevated in plasma during PEs. Immune mediators in plasma were compared between no PE (n = 17), prior PE (n = 11), and current PE (n = 6) groups using the Mann-Whitney test. An imputed value of ½ the LLOD was assigned for data points < LLOD (open symbol). *p ≤ 0.05, **p ≤ 0.01, and ***p ≤ 0.001.

Finally, we conducted the same comparisons in plasma. Concentrations of CCL2, IL-6, G-CSF, and IL-1RA were significantly higher in the current PE group compared to the no PE and prior PE groups (**Figure 4**). IL-1β was below the limit of detection in approximately half of the samples from no PE and prior PE groups, but was measurable in all but one sample from the current PE group. The concentration of IL-1β was comparable among samples from each group that were within the limits of detection.

### Extracellular NE activity, but not scavenging by myeloid cells, is increased during early CF PEs

**Figure 5:**
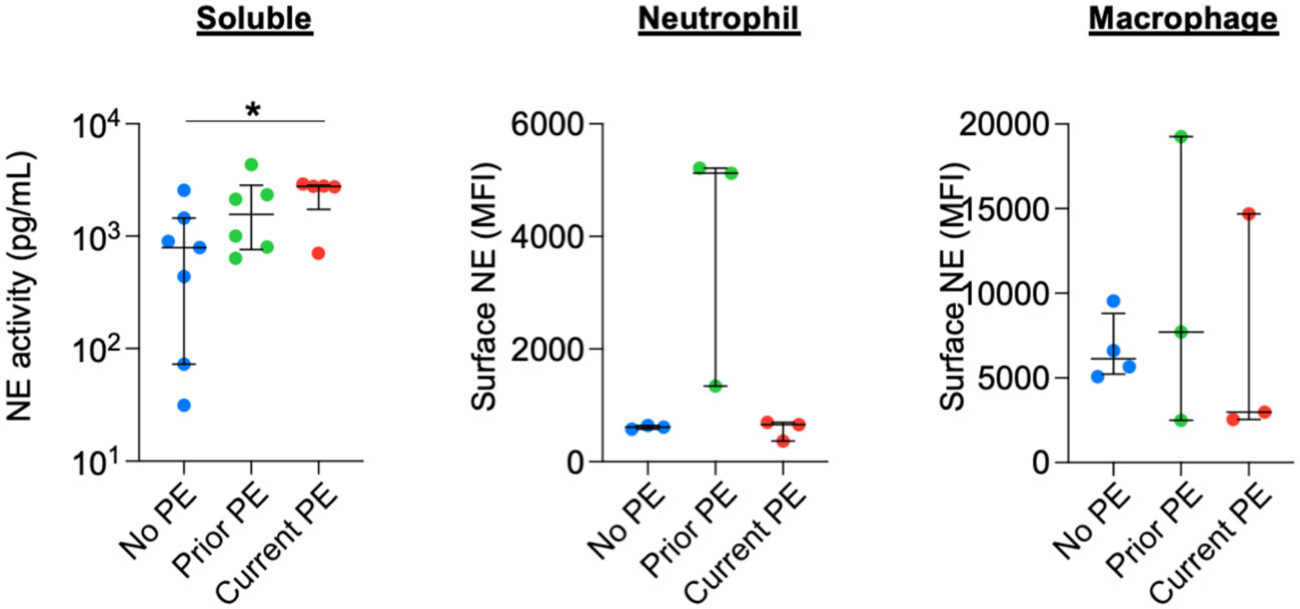
NE is not differentially secreted or scavenged during PEs. Soluble NE activity was quantified in BAL by FRET assay. Surface-bound NE on neutrophils and monocyte/macrophages from BAL was determined by flow cytometry. Comparisons between no PE (n = 7 for soluble, n = 3 for neutrophil, n = 4 for monocyte/macrophage), prior PE (n = 6 for soluble, n = 3 for neutrophil, n = 3 for monocyte/macrophage), and current PE (n = 5 for soluble, n = 3 for neutrophil, n = 3 for monocyte/macrophage) groups used using the Mann-Whitney test. *p ≤ 0.05.

We quantified the activity of soluble NE in BAL and compared it among the three types of study visits and observed increased NE activity in the current PE group compared to the no PE group. We also measured surface-bound NE on BAL neutrophils and monocytes/macrophages by flow cytometry but observed no significant differences between groups (**Figure 5**).

### Neutrophil frequency in circulation, but not the airway, increases during PE

**Figure 6:**
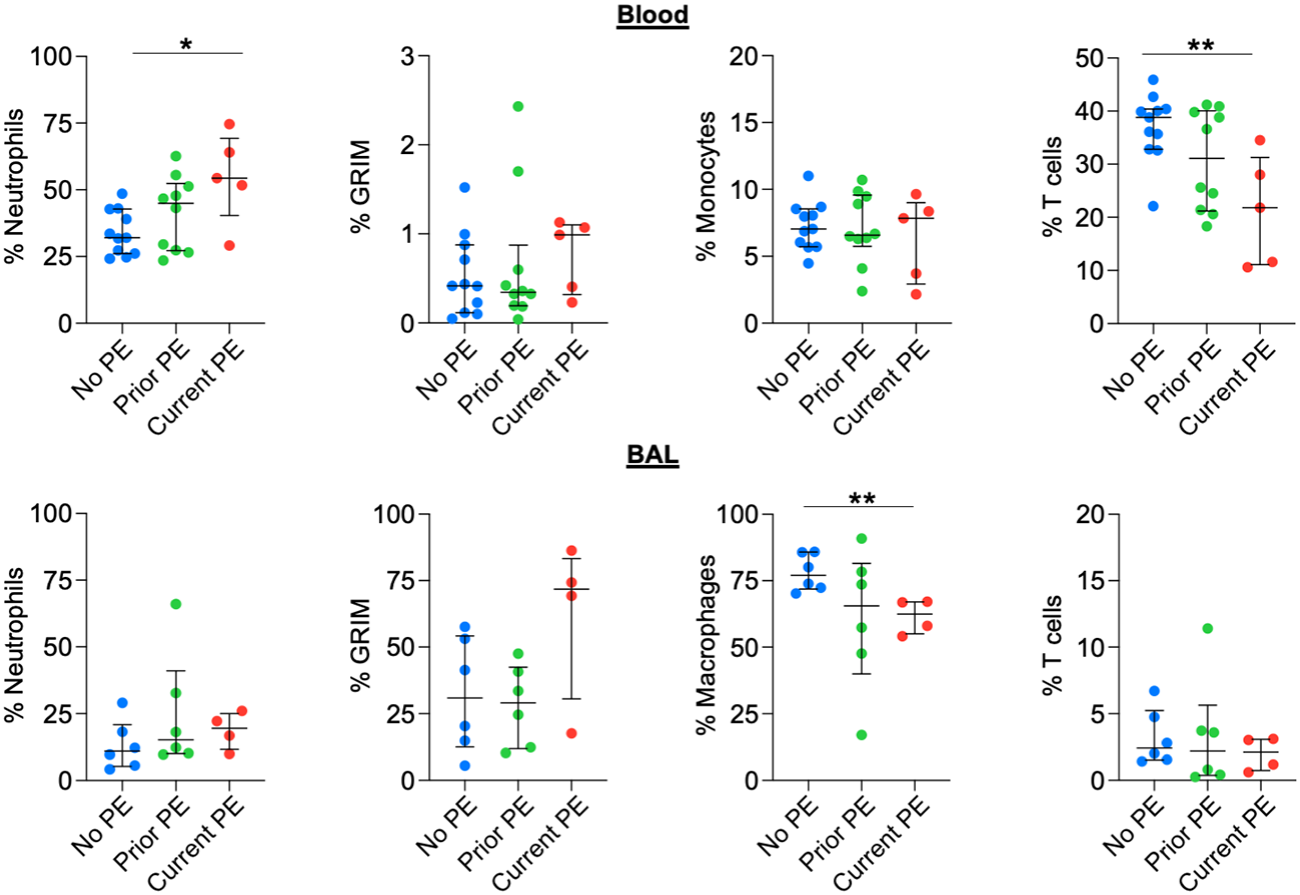
Neutrophil frequency in blood, but not BAL, increases during PE. The frequencies (out of total live leukocytes) of neutrophils, monocytes/macrophages, and T cells in blood and BAL, as well as that of BAL neutrophils displaying the GRIM phenotype were compared between no PE (n = 11 for blood, n = 6 for BAL), prior PE (n = 10 for blood, n = 6 for BAL), and current PE (n = 5 for blood, n = 4 for BAL) groups using the Mann-Whitney test. *p ≤ 0.05 and **p ≤ 0.01.

We used flow cytometry to identify neutrophil, monocyte/macrophage, and T-cell populations in blood and BAL and compared their frequencies out of total leukocytes in both fluids. We also calculated the proportion of neutrophils with the pathological GRIM phenotype defined as CD63^high^ and CD16^low^ (10), which have a major role in progression of CF lung disease (23). The frequency of neutrophils in blood was significantly higher in the current PE vs. no PE group, with a concomitant decrease in T-cell frequency, while there were no differences for proportion of blood monocytes. There were no significant differences in airway leukocyte frequencies between groups, although we noted a trend towards increased proportion of GRIM neutrophils in the current PE group (**Figure 6**).

### T cells modulate activating and inhibitory receptors during early CF PEs

**Figure 7:**
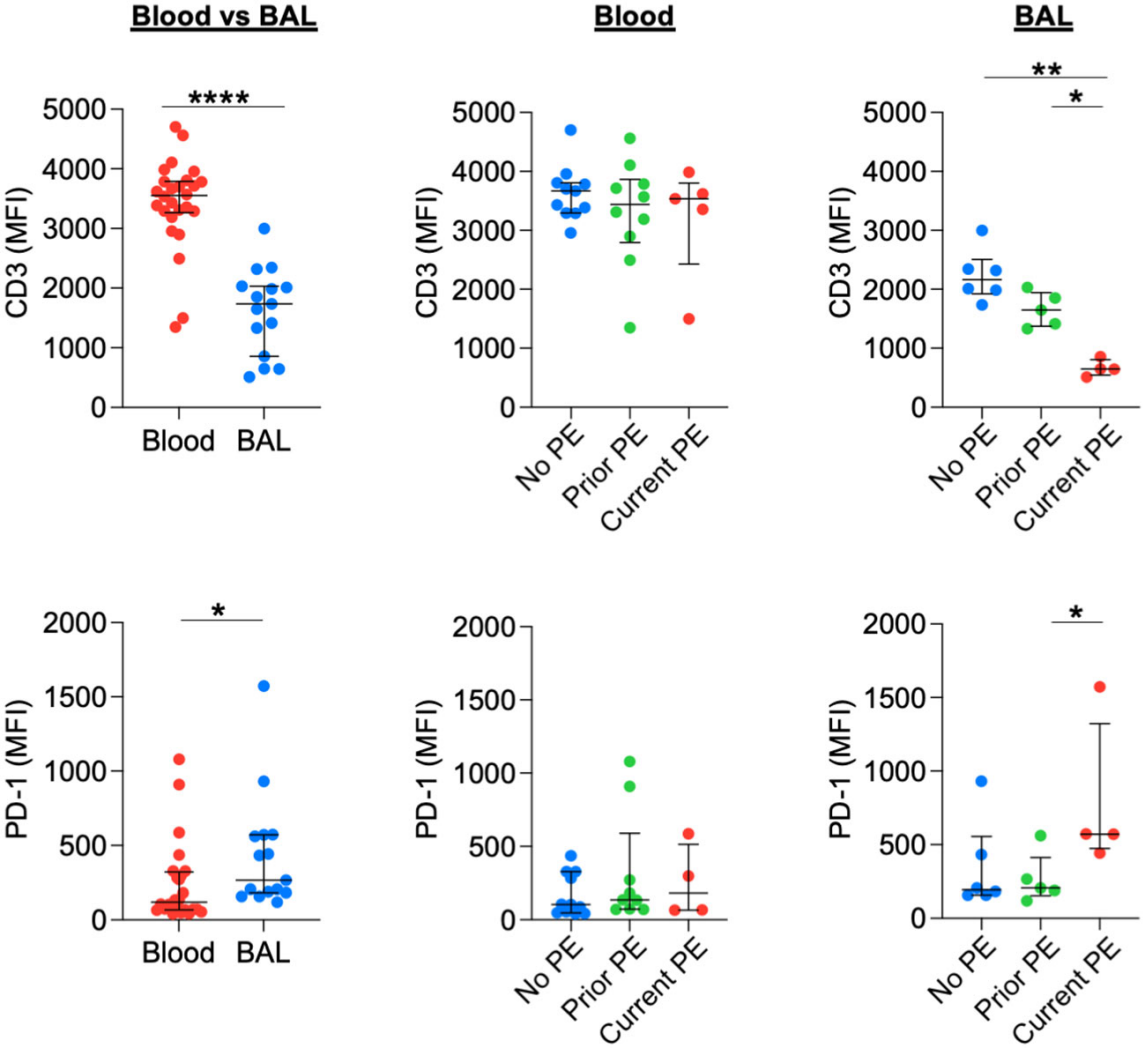
T cells downregulate CD3 and upregulate PD-1 during PEs. Surface expression of CD3 and PD-1 on T cells from blood and BAL was measured by flow cytometry and reported as median fluorescence intensity (MFI). Comparisons between blood (n = 26 for CD3 and n = 24 for PD-1) and BAL (n = 15 for CD3 and PD-1) used the Mann-Whitney test. No PE, prior PE, and current PE groups were also compared by the Mann-Whitney test. CD3: n = 11, 10, and 5 for no PE, prior PE, and current PE groups in blood and n = 6, 5, and 4 for no PE, prior PE, and current PE groups in BAL, respectively. PD-1: n = 11, 9, and 4 for no PE, prior PE, and current PE groups in blood and n = 6, 5, and 4 for no PE, prior PE, and current PE groups in BAL, respectively. *p ≤ 0.05, **p ≤ 0.01, and ****p ≤ 0.0001.

Previously, we showed that neutrophilic inflammation in CF airways, particularly their release of Arg1, contributes to inhibition of T-cell activity and their eventual exclusion from the airway lumen (13). Early in disease, however, T cells can still comprise 5-10% of total leukocytes obtained from BAL (18). We used flow cytometry to conduct a basic phenotypic analysis of T cells in blood vs, BAL and between the three groups of study visits reflecting PE history. BAL T cells showed a significant decrease in CD3 expression and a significant increase in PD-1 expression compared to their blood counterparts. There was no effect of PE history on expression of CD3 or PD-1 for blood T cells. However, CD3 expression was decreased, and PD-1 expression was increased for BAL T cells in the current PE group vs. prior PE group (**Figure 7**). We also investigated expression patterns of CD45 and PD-L1. CD45 expression did not differ in BAL compared to blood T cells. However, PD-L1 expression was significantly lower in the former. In addition, CD45 did not differ in blood but was significantly reduced on BAL T cells in the current PE group. PD-L1 expression was lower on T cells from both blood and BAL in the current PE group vs. both prior PE and no PE groups (**Figure S3**).

## Discussion

Through analysis of immune mediators and leukocyte subsets, this study provides new information on the coordination of immune responses in the airway during acute inflammatory reactions linked to PEs in young children with CF. With expanding use of highly effective CFTR modulatory therapy among younger patients with CF, including recent approval for elexacaftor/tezacaftor/ivacaftor for ages two and above, the natural history of the onset and progression of lung disease in young children is likely to change. This could significantly impact the age at which neutrophil predominance is established in the airways of young children and thereby change the rate of progression of lung disease over time. This evaluation of the airway immune cell interactions prior to the occurrence of chronic neutrophil influx and reduction in the proportion of airway macrophages and T cells provides key insights into the interactions between these cells in the context of repeated early life acute respiratory illnesses. When these acute respiratory illnesses are severe enough to be diagnosed as PE and require hospitalization, the differential changes in T-cell expression profile seen in BAL compared to blood suggests that these events could become the catalyst (or perhaps the trigger) for the pathological activation of neutrophils to a GRIM phenotype while simultaneously causing exhaustion of T cells through upregulation of PD-1 expression and reduction in CD3 expression. Further studies to track these changes in the post-modulator era will help to understand and develop better biomarkers for airway disease in young children with CF.

In this study, we collected blood and BAL fluid from young children with CF, who had either no history of hospitalization for PE, a prior PE event but were currently stable, or who were currently hospitalized for treatment of a PE. Half of the subjects who provided samples at stable clinic visits tested positive for airway colonization with pro-inflammatory pathogens but, as expected, the majority of subjects currently experiencing a PE tested positive for infection. Similarly, only half of subjects with no PE history had neutrophil frequencies of >10% in BAL, while the majority of subjects who had either prior or current hospitalizations had elevated BAL neutrophil frequencies. Considering that the overall comparison of neutrophil frequency in BAL was not different statistically between the no PE, prior PE and current PE groups, future studies including larger cohorts are needed to determine if PEs promote a higher threshold for neutrophil presence in the lung, as this may foreshadow impending chronic neutrophilic inflammation in the airways that is characteristic of established CF lung disease. Indeed, experiencing PEs is a recognized driving risk factor for occurrence of future PEs (17). While neutrophils were not yet dominant in the airways during this early stage of disease, we did observe a trend towards increasing frequency of neutrophils bearing the GRIM phenotype in BAL, suggesting that they are already beginning the process of reprogramming directing this pathological activity (10, 11) even if they do not yet constitute the dominant cell population therein.

We measured a panel of 20 immune mediators, including a) neutrophil mediators, b) monocyte/macrophage mediators, and c) anti-inflammatory mediators. The current PE group had significant increases in concentrations of cytokines from each subcategory in BAL, including IFN-γ and IL-10 which were largely unmeasurable in prior PE and no PE groups. The significant increase in the potent neutrophil chemoattractants CXCL1 and CXCL8 likely contribute to increasing neutrophil recruitment to the airways, but the concurrent increases in mediators related to monocyte/macrophage recruitment, such as CCL2, and resolution of inflammation, such as IL-10. This observation suggests that the airway immune response is not yet overwhelmed by neutrophilic inflammation, whereas later in the course of lung disease children with CF have long been known to exhibit elevated CXCL8 but lower IL-10 concentrations in the airways compared to healthy children (25). However, the increased concentration of plasma G-CSF and concurrent increase in frequency of circulating neutrophils during PEs suggests that inflammatory signaling feedback from the lungs may promote ongoing neutrophil recruitment to CF airways. This idea is supported by the modulation of osteoclast activity in response to PEs (6), and the decrease in total circulating leukocytes following intravenous antibiotic treatment (7). These previous studies show that even though CF may not be synonymous with systemic inflammation, changes in immune responses in the lungs may directly influence circulating cytokines and blood leukocyte subset distribution. While prior studies have documented an increase in inflammatory mediators in response to PEs and a decrease following treatment with antibiotics (4–8), the majority of these data are derived from adolescent and adult subjects, with limited data being reported from young children with CF who have yet to undergo persistent neutrophilic airway inflammation. Therapies aimed at blocking excessive neutrophil recruitment to the lung after resolution of a PE episode could potentially help to delay the onset of persistent neutrophilic airway inflammation in young children who experience these events frequently.

There are additional mediators of importance to lung disease not assessed in this study. For example, the IL-17 family of mediators (including IL-17 A, IL-17F and to a lesser extent IL-17 B, C D, and E) is a highly relevant target for CF lung disease given its role in combating bacterial and fungal pathogens, and its association with airway inflammation and PEs in CF (26). Additional studies are needed to address the relative levels of the IL-17 family of mediators in the neutrophil/T-cell crosstalk (reference) occurring during early CF PEs. Furthermore, it should be noted that our groupings of mediators as shown in **Figures 1**, **2** are a simplification and not representative of all sources of mediators (i.e., neutrophil, macrophage, epithelial, T-cell origins), nor of the breadth of their physiological roles and elicited responses. For example, CXCR3, the receptor for CXCL10 and CXCL11, is commonly associated with expression on T cells and lymphocytes but has been shown to be expressed on lung neutrophils from patients with acute respiratory distress syndrome (27).

Airway T cells represent an important line of defense against bacterial and viral pathogens (28). Although T-cell activity is inhibited by neutrophilic inflammation in the airway lumen of people with CF (13), Th17 cells and rare T-cell subsets are abundant in the submucosa (29). The Th17 pathway is actively engaged during pulmonary infections associated with CF and can further intensify neutrophilic inflammation (30), so understanding the level of T-cell engagement during PEs would be beneficial for improving immunomodulatory therapeutics which have shown promise but have yet to demonstrate definitive efficacy in treating CF lung disease (31). BAL T cells demonstrated phenotypic changes related to increased activation during PEs. CD3 is an important component of the T-cell receptor expressed at the cell surface with cytoplasmic tails that promote signal transduction. A decrease in CD3 is suggestive of activation, as TCR signaling has been shown to result both in degradation of CD3 at the cell surface (32) and reduced recycling (33).

Signaling through the PD-1 pathway is well characterized for its role in T-cell exhaustion during chronic viral infections and cancer (34), but expression of the receptor is induced on T and B lymphocytes upon cellular activation (35). When comparing T cells in BAL to those in blood, we observed decreased CD3 and increased PD-1 in the former, suggesting a heightened state of activation. It should be emphasized that samples for this study were collected from young children in the very early stages of CF lung disease, prior to established neutrophilic inflammation that represses airway T-cell activity (13). However, the observed decrease in CD3 and increase in PD-1 was most pronounced on BAL T cells from the current PE group, suggesting an active T-cell response to a bacterial and/or viral infection which is likely instigating the exacerbation. CD4+ T cells are important for orchestrating immune responses against bacterial infections, including in the respiratory tract (36). Arguably, the increase in PD-1 expression is unlikely to result in T cell exhaustion, as we previously showed that T cell inhibition in the airways is primarily due to cleavage of arginine by neutrophil-derived Arg1 rather than the PD-1 pathway (13). Moreover, as T cells increased PD-1 expression during PEs, the surface expression of its ligand PD-L1 was not increased on myeloid cells (data not shown) and was reduced on T cells. Assessing the level of airway T-cell activation during PE is complicated by the observation that CD45 (transmembrane phosphatase expressed in all leukocytes), which has roles for both positive and negative regulation of signaling in T cells (37), was reduced on T cells in the current PE group, but did not differ in blood vs. BAL (irrespective of PE status). CD45 expression was also reduced on BAL neutrophils and monocytes/macrophages during PEs. More work is needed to determine how CD45 modulates the activation poise in different leukocyte subsets during PEs compared to stable disease. In addition to T-cell activation, future studies should investigate how early life PEs influence development of CD69+ tissue-resident memory T cells (38), which may have important consequences for responding to pulmonary infections later in life. A more extensive subsetting of T cells including Th subsets should also be documented, which we were unable to perform here due to lack of material.

There are several limitations to our study. This was a single-center study, so validation of these findings in other cohorts would be beneficial. The overall number of study visits was small, in particular for those with a current PE. The ability to follow up on these findings with future sample collection will be limited by the declining number of PEs in general and in the availability of BAL, but these samples may still be collected at hospital visits for treatment of PE. As such, our reported findings are an important basis of future studies to make use of such rare and valuable samples. More frequent collection of airway samples will provide more data on the earliest inflammatory mechanisms and resolution of PEs and may be enabled by expanded use of induced sputum collection, which can be done in young children with CF and has many similarities as well some differences with BAL (18). Another limitation of this study is the lack of control groups, which prevents us to state decisively whether observed changes in CF PEs are CF-driven or PE-driven. Such control groups are difficult to enroll for BAL studies to compare to early CF. For example, diseased controls such as children with aerodigestive symptoms (39) or non-CF bronchiectasis (40) have complex etiologies that preclude simple comparisons to CF. On the flipside, BAL is very difficult to obtain from healthy control childrens, and prior data indicate that their BAL has completely different cell composition (41). More in-depth phenotyping of T cells, including determination of subset frequency and analysis of additional activation markers and effector molecules, as mentioned above, is also warranted. Studies with our local cohort were designed with a focus on myeloid cells in early CF lung disease and have recently been published (12, 18), but the findings presented here call for further investigation into adaptive immunity in the airways despite the eventual dominance by neutrophils and exclusion of T cells as CF lung disease progresses. Investigation into type I interferons are of particular importance for their role in initiating immune responses to viral as well as bacterial and fungal infections in the lung (42), all of which are highly relevant to CF lung disease. Our understanding of immune mediator production can be improved by mechanistic studies. While analysis of clinical samples as done here is translatable to patient health, we cannot discern the sources of mediators such as CXCL1 and CXCL8 (secreted by neutrophils and macrophages), or CXCL5 (secreted by various cell types) (43). Further studies may help improve our understanding of how the expression of these mediators differs between compartments.

In summary, we demonstrate that PEs in young children with CF are characterized by distinct immune mediator signatures but no significant phenotypic changes in airway neutrophils or monocytes/macrophages. However, we observed an increase in blood neutrophil frequency and slightly higher frequency of GRIM neutrophils in BAL, which may foreshadow the future mass-recruitment of neutrophils to the airway and their resulting contribution to progressive CF lung disease. Furthermore, we identified a signature for increased T-cell activation during early CF PEs reflected by reduced CD3 and increased PD-1 expression. These findings suggest that T cells may have a significant role in immune responses in the airway lumen early in CF lung disease, particularly during PEs, prior to the establishment of neutrophil dominance. These findings may also have implications for unsolved questions regarding CF lung disease such as the development of T-cell repertoires in the CF lung, and how this may relate to influence pathogen-induced respiratory illness in young children (44).

## Data availability statement

The datasets presented in this study can be found in online repositories. The names of the repository/repositories and accession number(s) can be found below: https://data.mendeley.com/datasets/rbm3ybxz54, 10.17632/rbm3ybxz54.2.

## Ethics statement

The studies involving humans were approved by Emory University Institutional Review Board. The studies were conducted in accordance with the local legislation and institutional requirements. Written informed consent for participation in this study was provided by the participants’ legal guardians/next of kin.

## Author contributions

VG, LG and RT contributed to conception and design of the study. GS and LG conducted clinical visits. VG conducted the experiments. VG, DG, GS and LG organized the database. VG, DG and LP performed the statistical analysis. VG wrote the first draft of the manuscript and JH supported revisions. LG and RT wrote sections of the manuscript. All authors contributed to the article and approved the submitted version.

## References

[1] Cystic Fibrosis Foundation. Cystic Fibrosis Foundation Patient Registry – Annual Data Report 2021. Bethesda, MD. (2021). Available at: https://www.cff.org/about-us/2021-annual-report.

[2] Goss CH. Acute pulmonary exacerbations in cystic fibrosis. Semin Respir Crit Care Med (2019) 40(6):792–803. doi: 10.1055/s-0039-1697975 31659730 PMC7528649

[3] Sanders DB Bittner RC Rosenfeld M Redding GJ Goss CH. Pulmonary exacerbations are associated with subsequent FEV1 decline in both adults and children with cystic fibrosis. Pediatr Pulmonol (2011) 46(4):393–400. doi: 10.1002/ppul.21374 20967845

[4] Solomon GM Frederick C Zhang S Gaggar A Harris T Woodworth BA. IP-10 is a potential biomarker of cystic fibrosis acute pulmonary exacerbations. PloS One (2013) 8(8):e72398. doi: 10.1371/journal.pone.0072398 23977293 PMC3745468

[5] McColley SA Stellmach V Boas SR Jain M Crawford SE. Serum vascular endothelial growth factor is elevated in cystic fibrosis and decreases with treatment of acute pulmonary exacerbation. Am J Respir Crit Care Med (2000) 161(6):1877–80. doi: 10.1164/ajrccm.161.6.9905022 10852760

[6] Shead EF Haworth CS Barker H Bilton D Compston JE. Osteoclast function, bone turnover and inflammatory cytokines during infective exacerbations of cystic fibrosis. J Cyst Fibros (2010) 9(2):93–8. doi: 10.1016/j.jcf.2009.11.007 20006563

[7] Colombo C Costantini D Rocchi A Cariani L Garlaschi ML Tirelli S. Cytokine levels in sputum of cystic fibrosis patients before and after antibiotic therapy. Pediatr Pulmonol (2005) 40(1):15–21. doi: 10.1002/ppul.20237 15858808

[8] Ordonez CL Henig NR Mayer-Hamblett N Accurso FJ Burns JL Chmiel JF. Inflammatory and microbiologic markers in induced sputum after intravenous antibiotics in cystic fibrosis. Am J Respir Crit Care Med (2003) 168(12):1471–5. doi: 10.1164/rccm.200306-731OC 12969869

[9] Houston CJ Taggart CC Downey DG. The role of inflammation in cystic fibrosis pulmonary exacerbations. Expert Rev Respir Med (2020) 14(9):889–903. doi: 10.1080/17476348.2020.1778469 32544353

[10] Tirouvanziam R Gernez Y Conrad CK Moss RB Schrijver I Dunn CE. Profound functional and signaling changes in viable inflammatory neutrophils homing to cystic fibrosis airways. Proc Natl Acad Sci USA (2008) 105(11):4335–9. doi: 10.1073/pnas.0712386105 PMC2393742 18334635

[11] Margaroli C Moncada-Giraldo D Gulick DA Dobosh B Giacalone VD Forrest OA. Transcriptional firing represses bactericidal activity in cystic fibrosis airway neutrophils. Cell Rep Med (2021) 2(4):100239. doi: 10.1016/j.xcrm.2021.100239 33948572 PMC8080108

[12] Margaroli C Horati H Garratt LW Giacalone VD Schofield C Dittrich AS. Macrophage PD-1 associates with neutrophilia and reduced bacterial killing in early cystic fibrosis airway disease. J Cyst Fibros (2022) 21(6):967–76. doi: 10.1016/j.jcf.2022.06.001 35732550

[13] Ingersoll SA Laval J Forrest OA Preininger M Brown MR Arafat D. Mature cystic fibrosis airway neutrophils suppress T cell function: evidence for a role of arginase 1 but not programmed death-ligand 1. J Immunol (2015) 194(11):5520–8. doi: 10.4049/jimmunol.1500312 PMC4433848 25926674

[14] Garratt LW Sutanto EN Ling KM Looi K Iosifidis T Martinovich KM. Matrix metalloproteinase activation by free neutrophil elastase contributes to bronchiectasis progression in early cystic fibrosis. Eur Respir J (2015) 46(2):384–94. doi: 10.1183/09031936.00212114 25929954

[15] Chmiel JF Davis PB. State of the art: why do the lungs of patients with cystic fibrosis become infected and why can't they clear the infection? Respir Res (2003) 4(1):8. doi: 10.1186/1465-9921-4-8 14511398 PMC203156

[16] Forrest OA Ingersoll SA Preininger MK Laval J Limoli DH Brown MR. Frontline Science: Pathological conditioning of human neutrophils recruited to the airway milieu in cystic fibrosis. J Leukoc Biol (2018) 104(4):665–75. doi: 10.1002/JLB.5HI1117-454RR PMC6956843 29741792

[17] VanDevanter DR Pasta DJ Konstan MW. Treatment and demographic factors affecting time to next pulmonary exacerbation in cystic fibrosis. J Cyst Fibros (2015) 14(6):763–9. doi: 10.1016/j.jcf.2015.02.007 PMC4561033 25754096

[18] Giacalone VD Moncada-Giraldo D Margaroli C Brown MR Silva GL Chandler JD. Pilot study of inflammatory biomarkers in matched induced sputum and bronchoalveolar lavage of 2-year-olds with cystic fibrosis. Pediatr Pulmonol (2022) 57(9):2189–98. doi: 10.22541/au.164376142.29605287/v1 35637404

[19] Gehrig S Mall MA Schultz C. Spatially resolved monitoring of neutrophil elastase activity with ratiometric fluorescent reporters. Angew Chem Int Ed Engl (2012) 51(25):6258–61. doi: 10.1002/anie.201109226 22555935

[20] Gehrig S Duerr J Weitnauer M Wagner CJ Graeber SY Schatterny J. Lack of neutrophil elastase reduces inflammation, mucus hypersecretion, and emphysema, but not mucus obstruction, in mice with cystic fibrosis-like lung disease. Am J Respir Crit Care Med (2014) 189(9):1082–92. doi: 10.1164/rccm.201311-1932OC 24678594

[21] Dittrich AS Kuhbandner I Gehrig S Rickert-Zacharias V Twigg M Wege S. Elastase activity on sputum neutrophils correlates with severity of lung disease in cystic fibrosis. Eur Respir J (2018) 51(3):1701910. doi: 10.1183/13993003.01910-2017 29545279

[22] Tirouvanziam R Diaz D Gernez Y Laval J Crubezy M Makam M. An Integrative Approach for Immune Monitoring of Human Health and Disease by Advanced Flow Cytometry Methods. In: Advanced Optical Flow Cytometry: Methods and Disease Diagnoses, 1st ed. (Weinheim, Germany: Wiley-VCH Verlag GmbH & Co. KGaA). (2011). p. 333–62.

[23] Margaroli C Garratt LW Horati H Dittrich AS Rosenow T Montgomery ST. Elastase exocytosis by airway neutrophils is associated with early lung damage in children with cystic fibrosis. Am J Respir Crit Care Med (2019) 199(7):873–81. doi: 10.1164/rccm.201803-0442OC PMC6444666 30281324

[24] Gangell C Gard S Douglas T Park J de Klerk N Keil T. Inflammatory responses to individual microorganisms in the lungs of children with cystic fibrosis. Clin Infect Dis (2011) 53(5):425–32. doi: 10.1093/cid/cir399 21844026

[25] Osika E Cavaillon JM Chadelat K Boule M Fitting C Tournier G. Distinct sputum cytokine profiles in cystic fibrosis and other chronic inflammatory airway disease. Eur Respir J (1999) 14(2):339–46. doi: 10.1183/09031936.99.14233999 10515411

[26] Gurczynski SJ Moore BB. IL-17 in the lung: the good, the bad, and the ugly. Am J Physiol Lung Cell Mol Physiol (2018) 314(1):L6–L16. doi: 10.1152/ajplung.00344.2017 28860146 PMC6048455

[27] Ichikawa A Kuba K Morita M Chida S Tezuka H Hara H. CXCL10-CXCR3 enhances the development of neutrophil-mediated fulminant lung injury of viral and nonviral origin. Am J Respir Crit Care Med (2013) 187(1):65–77. doi: 10.1164/rccm.201203-0508OC 23144331 PMC3927876

[28] Muruganandah V Sathkumara HD Navarro S Kupz A. A systematic review: The role of resident memory T cells in infectious diseases and their relevance for vaccine development. Front Immunol (2018) 9:1574. doi: 10.3389/fimmu.2018.01574 30038624 PMC6046459

[29] Tan HL Regamey N Brown S Bush A Lloyd CM Davies JC. The Th17 pathway in cystic fibrosis lung disease. Am J Respir Crit Care Med (2011) 184(2):252–8. doi: 10.1164/rccm.201102-0236OC PMC3381840 21474644

[30] Dubin PJ McAllister F Kolls JK. Is cystic fibrosis a TH17 disease? Inflamm Res (2007) 56(6):221–7. doi: 10.1007/s00011-007-6187-2 17607545

[31] Giacalone VD Dobosh BS Gaggar A Tirouvanziam R Margaroli C. Immunomodulation in cystic fibrosis: why and how? Int J Mol Sci (2020) 21(9):3331. doi: 10.3390/ijms21093331 32397175 PMC7247557

[32] Valitutti S Muller S Salio M Lanzavecchia A. Degradation of T cell receptor (TCR)-CD3-zeta complexes after antigenic stimulation. J Exp Med (1997) 185(10):1859–64. doi: 10.1084/jem.185.10.1859 PMC2196323 9151711

[33] Liu H Rhodes M Wiest DL Vignali DA. On the dynamics of TCR:CD3 complex cell surface expression and downmodulation. Immunity (2000) 13(5):665–75. doi: 10.1016/S1074-7613(00)00066-2 11114379

[34] Pauken KE Torchia JA Chaudhri A Sharpe AH Freeman GJ. Emerging concepts in PD-1 checkpoint biology. Semin Immunol (2021) 52:101480. doi: 10.1016/j.smim.2021.101480 34006473 PMC8545711

[35] Agata Y Kawasaki A Nishimura H Ishida Y Tsubata T Yagita H. Expression of the PD-1 antigen on the surface of stimulated mouse T and B lymphocytes. Int Immunol (1996) 8(5):765–72. doi: 10.1093/intimm/8.5.765 8671665

[36] Eddens T Kolls JK. Host defenses against bacterial lower respiratory tract infection. Curr Opin Immunol (2012) 24(4):424–30. doi: 10.1016/j.coi.2012.07.005 PMC3971433 22841348

[37] Courtney AH Shvets AA Lu W Griffante G Mollenauer M Horkova V. CD45 functions as a signaling gatekeeper in T cells. Sci Signal (2019) 12(604):eaaw8151. doi: 10.1126/scisignal.aaw8151 31641081 PMC6948007

[38] Takamura S Yagi H Hakata Y Motozono C McMaster SR Masumoto T. Specific niches for lung-resident memory CD8+ T cells at the site of tissue regeneration enable CD69-independent maintenance. J Exp Med (2016) 213(13):3057–73. doi: 10.1084/jem.20160938 PMC5154946 27815325

[39] Duncan DR Cohen A Golden C Lurie M Mitchell PD Liu E. Gastrointestinal factors associated with risk of bronchiectasis in children. Pediatr Pulmonol (2023) 58(3):899–907. doi: 10.1002/ppul.26276 36510759 PMC9957932

[40] Beckeringh NI Rutjes NW van Schuppen J Kuijpers TW. Noncystic fibrosis bronchiectasis: evaluation of an extensive diagnostic protocol in determining pediatric lung disease etiology. Pediatr Allergy Immunol Pulmonol (2019) 32(4):155–62. doi: 10.1089/ped.2019.1030 PMC7057054 32140286

[41] Shanthikumar S Ranganathan SC Saffery R Neeland MR. Mapping pulmonary and systemic inflammation in preschool aged children with cystic fibrosis. Front Immunol (2021) 12:733217. doi: 10.3389/fimmu.2021.733217 34721395 PMC8554310

[42] Makris S Paulsen M Johansson C. Type I interferons as regulators of lung inflammation. Front Immunol (2017) 8:259. doi: 10.3389/fimmu.2017.00259 28344581 PMC5344902

[43] Deng J Jiang R Meng E Wu H. CXCL5: A coachman to drive cancer progression. Front Oncol (2022) 12:944494. doi: 10.3389/fonc.2022.944494 35978824 PMC9376318

[44] Wang EE Prober CG Manson B Corey M Levison H. Association of respiratory viral infections with pulmonary deterioration in patients with cystic fibrosis. N Engl J Med (1984) 311(26):1653–8. doi: 10.1056/NEJM198412273112602 6504106

