# Supplementary material for: Pulmonary exacerbations in early cystic fibrosis lung disease are marked by strong modulation of CD3 and PD-1 on luminal T cells
Source: Front Immunol. 2023 Sep 21;14:1194253. doi: 10.3389/fimmu.2023.1194253 (PMC10551126; doi:10.3389/fimmu.2023.1194253)
Supplement: Supplementary file 1 [file DataSheet_1.pdf]

Supplementary Material

Pulmonary exacerbations in early cystic fibrosis lung disease are marked by strong modulation of CD3 and PD-1 on luminal T cells

Vincent D. Giacalone[1,2], Diego Moncada-Giraldo[1,2], George L. Silva[1,2], Justin Hosten[3], Limin Peng[4], Lokesh Guglani[1,2§,] Rabindra Tirouvanziam[1,2,3§*,] on behalf of the IMPEDE-CF Program

[1]Department of Pediatrics, Emory University, Atlanta, GA, USA

[2]Center for CF and Airways Disease Research, Children's Healthcare of Atlanta, Atlanta, GA, USA

[3]Wallace H. Coulter Department of Biomedical Engineering, Georgia Institute of Technology and Emory University, Atlanta, GA, USA

[4]Department of Biostatistics and Bioinformatics, Emory University School of Public Health, Atlanta, GA, USA.
[§] Co-senior authors.

*** Correspondence:**
Rabindra Tirouvanziam, PhD
tirouvanziam@emory.edu

Table S1. Sample collection totals. 39 clinic visits were conducted for collection of blood and/or BAL. Not all samples could be obtained due to logistical or biological limitations.

Subject	Blood		BAL		
	Whole blood (mL)	Plasma (mL)	Total volume (mL)	Live leukocytes	Cells/µL
1	4.8	2	3	3.60E+05	120
1, visit 2					
1, visit 3	2.45	1			
2	3.5	1.4	8.36	1.90E+05	23
2, visit 2	2.4	0.865			
2, visit 3	3	1			
3	3.2	1.7	10.5	1.50E+06	143
4					
5	3.4	1.4	4.2	4.40E+05	105
6	3.5	1.8			
7	3.1	1.2	2	2.00E+06	1000
7, visit 2	4.2	2.2			
7, visit 3	3.5	1.7	2.7	2.00E+04	8
8	4.5	2.3	1.4	2.70E+06	1929
9	3.5	1.5			
10	2.8	1.1			
11	3.5	1.2	1.2	9.00E+04	75
11, visit 2	2.5	0.92			
11, visit 3	3	1			
12	2.5	0.9			
13	3.5	1.2	2	9.30E+04	47
13, visit 2	2.43	0.95	2.4	4.50E+06	1875
13, visit 3					
14	2.5	1			
15	3.6	2	1.35	1.08E+07	8000
16	3.4	1.3			
17					
17, visit 2	2.6	1	0.35	4.20E+05	1200
18	2.05	1	4.45	5.00E+05	112

18, visit 2	1.3	0.6			
19	2.8	1.34	3.19	7.20E+05	226
20	3.1	1.4			
21	2.4	0.84			
22	2.9	1.22	3.8	2.40E+05	63
23	2.9	1.2	3.7	5.30E+05	143
23, visit 2	3	1.15			
24	2.8	1.9	3.2	7.30E+05	228
25	2.4	1.2			
26	3	1.5	3.5	1.60E+05	46

Table S2. Total assays performed for blood and BAL samples. Soluble mediator assays and flow cytometry analysis were conducted for blood and BAL samples where indicated by an "X".

**Subject**	**Clinical data**	**Soluble mediators**			**Flow cytometry**			
		Plasma Cytokines	BAL cytokines	BAL NE	Blood P14	Blood P15	BAL P14	BAL P15
1	X	X	X	X	X	X	X	X
1, visit 2	X							
1, visit 3	X	X						
2	X	X	X	X	X	X	X	X
2, visit 2	X	X			X	X		
2, visit 3	X	X						
3	X	X	X	X	X	X	X	X
4	X							
5	X	X	X	X	X	X	X	X
6	X	X			X	X		
7	X		X	X	X	X	X	X
7, visit 2	X	X						
7, visit 3	X	X	X	X	X	X		X
8	X	X	X	X	X	X	X	X
9	X	X			X	X		
10	X	X			X	X		
11	X	X	X	X	X	X		X
11, visit 2	X	X				X		
11, visit 3	X	X						
12	X	X			X	X		
13	X	X	X	X	X	X		X
13, visit 2	X	X	X	X	X			X
13, visit 3	X							
14	X	X			X	X		
15	X	X	X	X				
16	X	X			X	X		
17	X							
17, visit 2	X	X	X	X	X	X	X	X
18	X	X	X	X	X	X		X
18, visit 2	X	X						

19	X	X	X	X				X
20	X	X				X		
21	X	X						
22	X	X	X	X	X	X	X	X
23	X	X	X	X	X	X	X	X
23, visit 2	X	X						
24	X	X	X	X	X	X	X	X
25	X	X			X	X		
26	X	X	X	X				

Table S3. Flow cytometry staining panels. Two staining panels of 10 colors each were designed and used for the duration of this study. Each panel contained a set of common backbone parameters in addition to three channels with panel-specific parameters.

Color	Stain	Vendor	Catalog	Clone
**Backbone**				
PB	Calcein violet	Thermo Fisher	C34858	n/a
PB	CD41a	Biolegend	303714	HIP8
PB	CD3	Biolegend	300330	HIT3a
BV605	CD45	Biolegend	304042	HI30
BV650	CD63	Biolegend	353026	H5C6
PER CP CY 5.5	CD16	Biolegend	302028	3G8
PE	CD66b	Biolegend	305106	G10F5
PE CY7	CD115	Biolegend	347308	9-4D2-1E4
AF700	Siglec-8	R&D Systems	FAB7975N	837535
**Panel 14**				
FITC	NE	R&D Systems	IC91671G-100UG	950317
APC	CD36	Biolegend	336208	5-271
APC CY7	CD304	Biolegend	354524	12C2
**Panel 15**				
FITC	PD-1	Biolegend	329904	EH12.2H7
APC	PD-L1	Biolegend	329708	29E.2A3
APC CY7	EGFR (unconjugated)	Biolegend	352902	AY13
	APC Cy7 conjugation kit	Novus Biologicals	765-0010	n/a

Table S4. Expanded demographics. Abbreviations: W, White; F, Female; M, Male; MSSA, methicillin-susceptible *S. aureus*; MRSA, methicillin-resistant *S. aureus*.

Subject #	PE status	Age (months)	Sex	Race/ Ethnicity	Mutation 1	Mutation 2	Bacterial infection	Viral infection
**1**	No PE	33	M	W	F508del	E585X	MSSA	
**1, visit 2**	No PE	45	M	W	F508del	E585X	MSSA	
**1, visit 3**	No PE	65	M	W	F508del	E585X	MSSA	
**2**	No PE	26	M	W	F508del	F508del	MSSA	
**2, visit 2**	No PE	40	M	W	F508del	F508 del		
**2, visit 3**	No PE	58	M	W	F508del	F508 del		
**3**	No PE	35	F	W	F508del	F508del		
**4**	Prior PE	48	M	W	F508del	1717-1 G>A	*S. marcescens*	
**5**	Prior PE	35	M	W	F508del	R553X		
**6**	Current PE	102	F	W	F508del	F508del	MRSA	
**7**	Current PE	23	M	W	F508del	621+1G>T	MRSA	Parainfluenza 4 PCR
**7, visit 2**	Current PE	25	M	W	F508del	F508del	MRSA	Rhinovirus, Influenza A PCR
**7, visit 3**	Prior PE	27	M	W	F508del	F508del	MRSA	
**8**	Current PE	17	M	W	F508del	F508del	MRSA	
**9**	No PE	15	F	W	F508del	F508del		
**10**	No PE	16	M	W	F508del	F508del	MSSA	
**11**	No PE	36	F	W	F508del	5T with TG11	MRSA	
**11, visit 2**	No PE	49	F	W	F508del	5T with TG11		
**11, visit 3**	No PE	64	F	W	F508del	5T with TG11	MRSA	
**12**	Prior PE	75	F	W	M1101K	2789 +5G>A		
**13**	Prior PE	25	M	Mixed	3120+1 G>A	c.3468+2dupT		
**13, visit 2**	Prior PE	30	M	Mixed	3120+1 G>A	c.3468+2dupT		

**13, visit 3**	Prior PE	35	M	Mixed	3120+1 G>A	c.3468+2dupT	MSSA	
**14**	Prior PE	68	M	W	F508del	1898+1G>A	MSSA	
**15**	Current PE	61	M	W	F508del	F508del		
**16**	No PE	51	F	Latina	F508del	D1152H	MSSA	
**17**	Current PE	53	M	W	F508del	621+1G>T	MSSA	
**17, visit 2**	Current PE	61	M	W	F508del	621+1G>T	MSSA	Rhinovirus
**18**	No PE	26	F	W	F508del	F508del	MSSA	
**18, visit 2**	No PE	44	F	W	F508del	F508 del		
**19**	Current PE	10	M	W	F508del	F508del		
**20**	Prior PE	70	F	W	F508del	F508del		
**21**	No PE	77	M	W	F508del	1898+1G>A		
**22**	No PE	23	M	W	G542X	c.del_exon25_exon26		
**23**	Prior PE	28	M	W	F508del	F508 del		
**23, visit 2**	Prior PE	50	M	W	F508del	F508 del	MRSA	
**24**	Prior PE	31	M	W	F508del	R553X		
**25**	Prior PE	64	M	W	F508del	5T-TG13	MRSA	
**26**	No PE	83	M	W	F508del	2789+2insA		

Supplementary Figures

Figure S1. Dot plot representation of infection status and neutrophil frequency in BAL. Infection and airway neutrophil frequency data from Table 2 is shown with one dot corresponding to one subject.

Figure S2. Flow cytometry gating strategy. Cells from blood and BAL were stained for analysis by flow cytometry (represenative sample shown here for illustrative purposes). All major cell populations in both sample types were identified by gating in Flowjo. (A) The same upstream gates were applied to all blood and BAL samples beginning with a time gate to exclude air bubbles or other errors with sample acquisition. Debris and red blood cells were excluded by gating on CD45+ events. Single cells were identified using forward scatter-area and forward scatter-height. After gating on live cells, individual cell populations could then be identified. (B) In blood, platelet-free eosinophil and neutrophil populations were selected by gating on CD41a- events. Among platelet-free neutrophils, the A1 and A2 (GRIM) subpopulations were identified using CD16 and CD63. Among PBMCs, monocytes were gated on as CD115+ and having higher side scatter. Platelet-free monocytes were then selected. Platelet-free T cells and CD16+ NK cells were gated from the lymphocyte population. (C) Following the live gate in BAL samples, CD66b and CD45 were used for gating on neutrophils, while CD66b and CD115 were applied for macrophages and lymphocytes followed by additional gating steps. Neutrophils were again divided into the A1 and A2 (GRIM) subpopulations, while T cells were selected from $CD115^{low}$ events following identification of the airway monocyte/macrophage population.

Figure S3. T cells downregulate CD45 and PD-L1 during PEs. Surface expression of CD45 and PD-L1 on T cells from blood and BAL was measured by flow cytometry and reported as median fluorescence intensity (MFI). Comparisons between blood (n = 26 for CD45 and n = 24 for PD-L1) and BAL (n = 15 for CD45 and PD-L1) used the Mann-Whitney test. No PE, prior PE, and current PE groups were compared by the Mann-Whitney test. CD45: n = 11, 10, and 5 for no PE, prior PE, and current PE groups in blood and n = 6, 5, and 4 for no PE, prior PE, and current PE groups in BAL, respectively. PD-L1: n = 11, 9, and 4 for no PE, prior PE, and current PE groups in blood and n = 6, 5, and 4 for no PE, prior PE, and current PE groups in BAL, respectively. $*p \leq 0.05$, $**p \leq 0.01$, and $****p \leq 0.0001$.
