# Supplementary figures and images for: Pulmonary exacerbations in early cystic fibrosis lung disease are marked by strong modulation of CD3 and PD-1 on luminal T cells
Source: Front Immunol. 2023 Sep 21;14:1194253. doi: 10.3389/fimmu.2023.1194253 (PMC10551126; doi:10.3389/fimmu.2023.1194253)

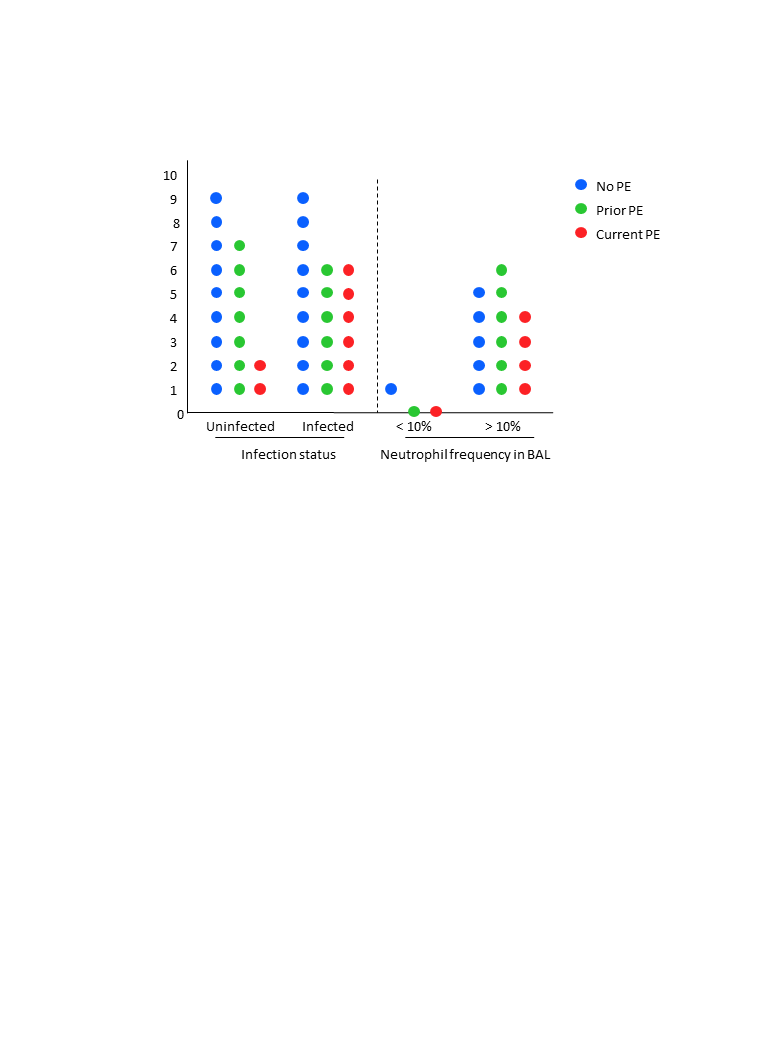

Supplement: Supplementary file 2 [file Image_1.tiff]

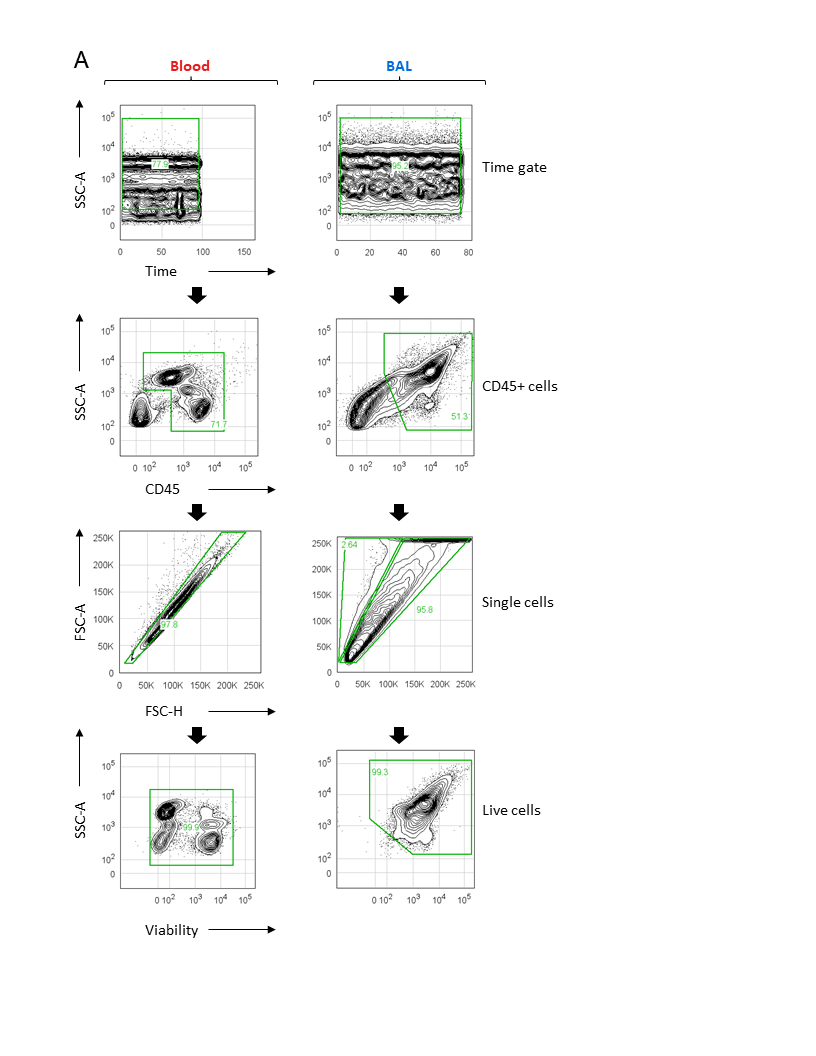

Supplement: Supplementary file 3 [file Image_2.png]

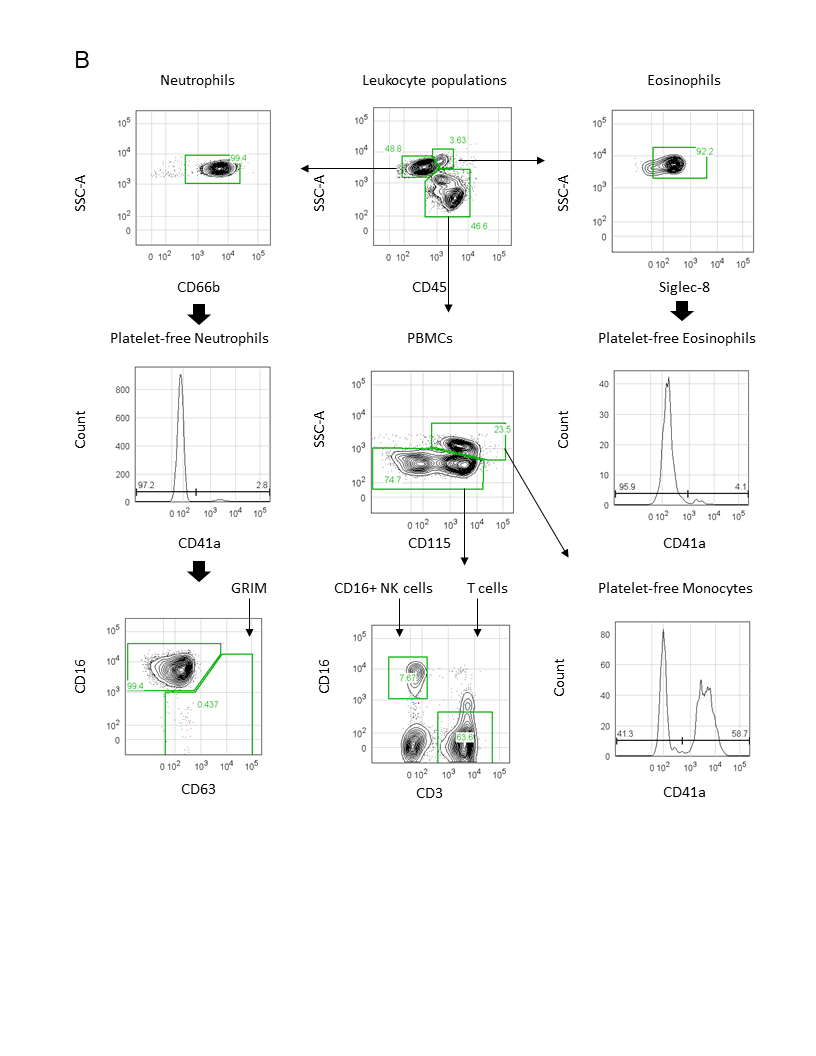

Supplement: Supplementary file 4 [file Image_3.png]

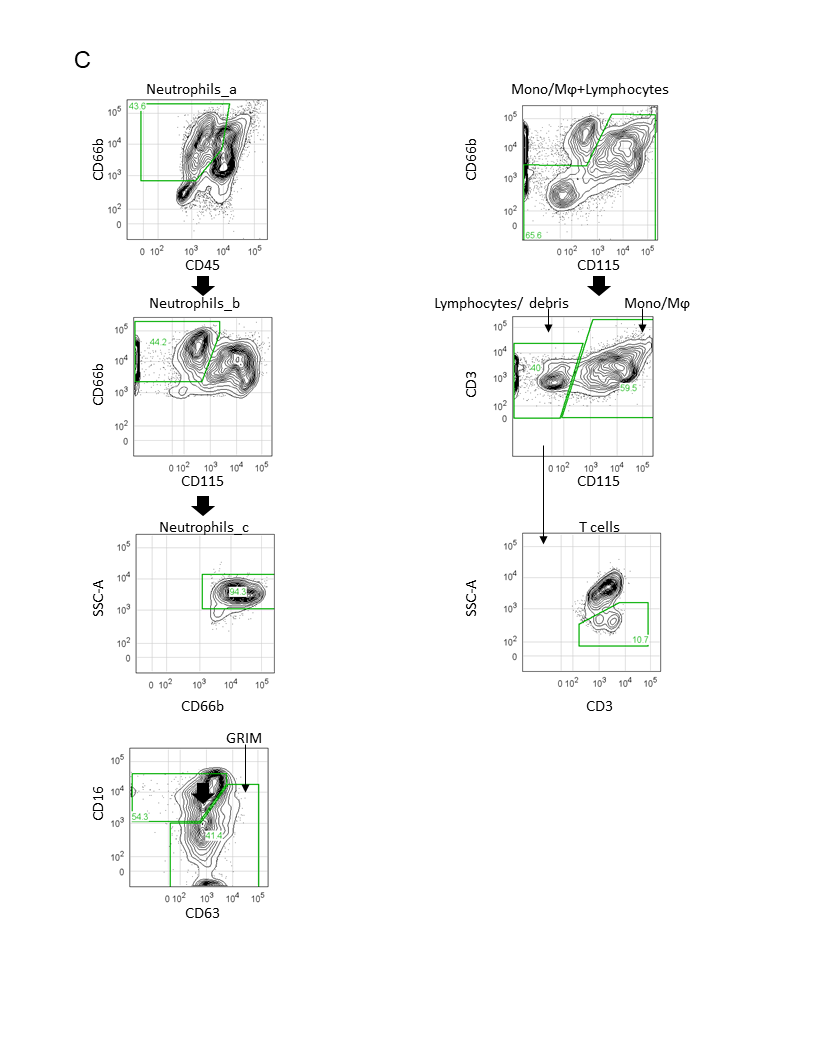

Supplement: Supplementary file 5 [file Image_4.png]

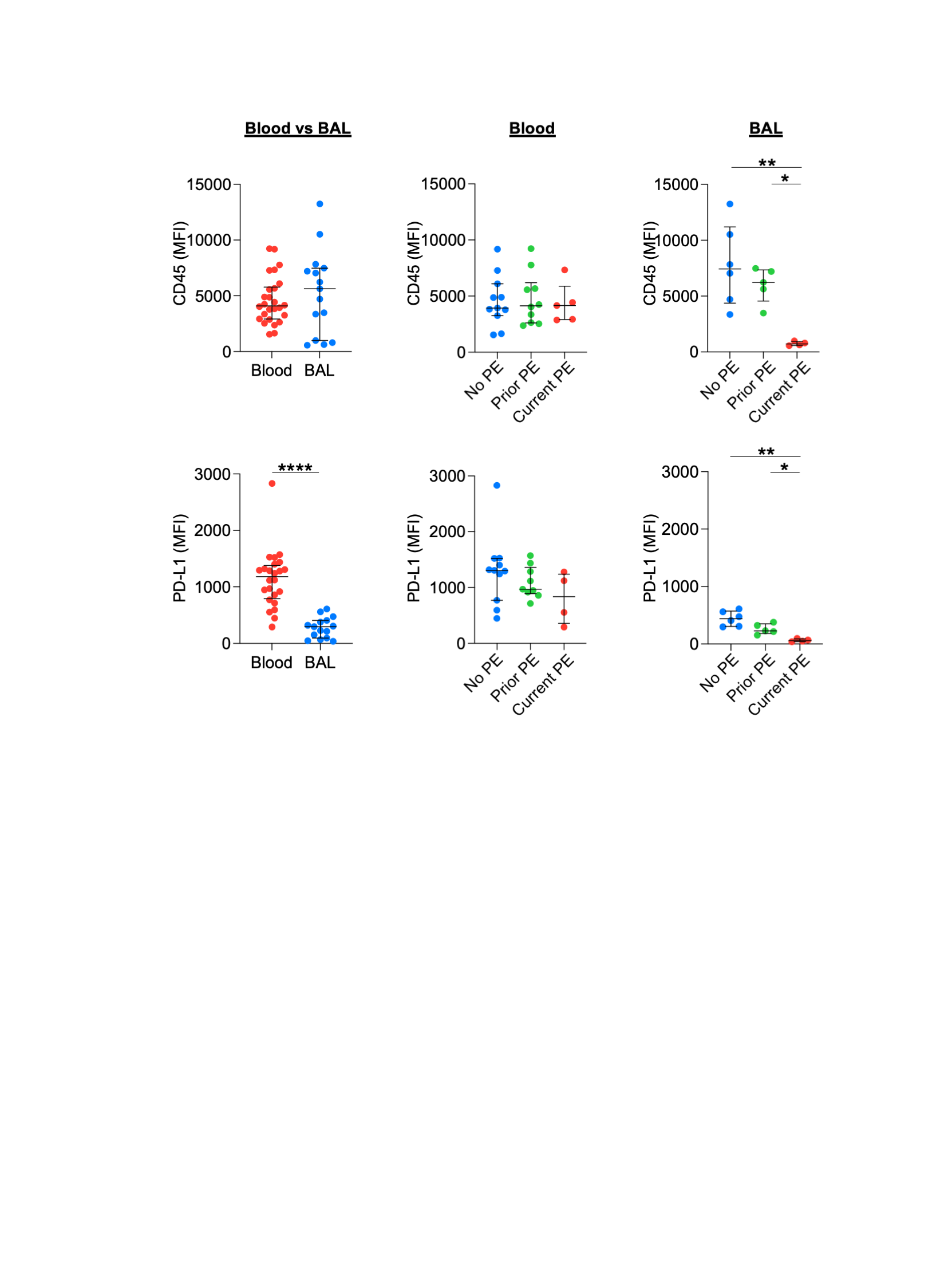

Supplement: Supplementary file 6 [file Image_5.tiff]
